# The Fingerprint Identification of Asphalt Aging Based on ^1^H-NMR and Chemometrics Analysis

**DOI:** 10.3390/ma15196825

**Published:** 2022-10-01

**Authors:** Wenxin Wu, Chenlong Wang, Pinhui Zhao, Linyan Xiu, Liang Fan, Fei Bi, Xiaoqing Song, Xu Zhou

**Affiliations:** 1 School of Transportation Engineering, Shandong Jianzhu University, Jinan 250101, China; 2 Shandong Runxingcheng Road Construction Materials Research and Development Center, Yantai Highway Business Development Center, Yantai 265600, China; 3 Shandong Institute of Transportation Science, Jinan 250102, China

**Keywords:** asphalt, aging, ^1^H-NMR, fingerprint identification, chemometrics analysis

## Abstract

In this study, the chemical structure of asphalt aging was analyzed and identified based on ^1^H-NMR quantitative technology and chemometrics analysis. The characteristic full component information of 30 samples before and after aging from 5 different oil sources was measured by ^1^H-NMR, and the results were converted into a data matrix. This study used PCA, HAC, OPLS-DA, and Fisher discriminant analysis to evaluate the change rules of the chemical composition of asphalt from different oil sources after aging. The results showed that the ^1^H-NMR spectra of 30 asphalt samples were very similar, and hydrogen could be divided into 4 categories according to the chemical shift: H_A_, H_α_, H_β_, and H_γ_. The shapes of ^1^H-NMR of asphalt samples from different oil sources showed slight differences, while the shapes of the ^1^H-NMR spectra of asphalt samples with different aging degrees from the same oil source was basically the same. The results of PCA and HAC analysis showed that the samples of the same asphalt and asphalt with similar oil sources before and after aging were still in the same category, and the spatial distance was very close, while the spatial distance of asphalts from different oil sources was very different. The Fisher discriminant function established by PCA and HAC can be used to distinguish asphalt samples from different oil sources with an accuracy of up to 100%.

## 1. Introduction

Asphalt is a by-product of the petroleum industry that is widely used in pavement construction. During pavement use, asphalt undergoes a series of complex physical and chemical changes, such as volatilization, oxidation, and condensation, which make the asphalt hard and brittle, and further lead to the deterioration of the pavement structure, such as fatigue, cracking, and moisture damage. This process is called asphalt aging [1,2]. The thin film oven test (TFOT), rolling thin film oven test (RTFOT), and pressurized aging vessel (PAV) are usually used in the laboratory to simulate short-term aging and long-term aging [3]. At present, much research work has been carried out on asphalt aging based on simulating asphalt aging in the laboratory, and many testing techniques and performance indicators have been proposed for evaluating the degree of asphalt aging [4,5]. The most extensive research on asphalt aging is on the changes in physical properties. Many scholars have studied the influence of asphalt aging on pavement performance by conducting aging experiments on asphalt and analyzing the changes in physical performance indexes such as penetration, softening point, ductility, viscosity, creep stiffness, and dynamic viscoelasticity [6]. After significant amounts of research, a deeper understanding of the changes of physical indexes of performance of aged asphalt now exists. It is generally believed that the aging performance of different asphalts is basically the same. That is, with increasing aging time, the penetration and ductility of asphalts decrease, while the softening point, viscosity, complex shear modulus, and creep stiffness gradually increase [7]. Additionally, the elasticity of asphalt is enhanced, the temperature sensitivity is weakened, and the fatigue cracking resistance becomes poor after aging, thus shortening the service life of the pavement [8,9].

Although the aging performance of asphalt varies in the same way, the aging resistance and durability of different asphalts greatly vary. This is the external manifestation of the chemical composition, molecular structure, and transformation of asphalt. Therefore, it is of great significance to study the influence of the chemical composition and structure on the aging performance of asphalt in order to reveal its aging law and mechanism. With the progress in instrumental analysis technology, the research on asphalt aging has gradually shifted from the changes in macroscopic physical indexes to the characterization of the microstructure. Many scholars have studied the changes in the microscopic properties of asphalt before and after aging, by means of chemical microscopic characterization such as the infrared spectrum, gel chromatography, elemental analysis, thermogravimetry, and mass spectrometry, in order to explore its aging mechanism [10,11,12,13,14]. For example, gel permeation chromatography (GPC) can be used to detect and analyze the decreases in small molecular substances and the increases in large molecular substances after asphalt aging, and its test results can be used to predict the aging degree of asphalt [15,16]. Many scholars use Fourier transform infrared spectroscopy (FTIR) to quantitatively analyze the aging degree of asphalt at the molecular level. The results showed that carbonyl index (CI) is suitable for evaluating the aging effect of base asphalt, and the butadiene index (BI) is suitable for evaluating the aging effect of modified asphalt [17,18]. However, FTIR is only suitable for the study of structural changes in the same asphalt after aging. In addition, fluorescent spectral intensity, spectrophotometric changes, and fluorescent images can also be used as references to evaluate the degree of asphalt aging [19]. Different compounds in asphalt have completely different chemical properties because of asphalt’s complex composition, so it is not possible to determine all of them in a single chemical analysis. However, the analytical testing techniques mentioned above usually focus on a few major compounds in the determination of the chemical composition of asphalt, which may mislead its identification and evaluation. In addition, there is no report on the differences in chemical composition of asphalt from different oil sources after aging, so it is necessary to conduct nontargeted chemical fingerprint analysis of aging asphalt and analyze asphalt samples from different oil sources in order to study the differences or similarities in chemical composition of asphalt after aging.

Compared with other technologies, nuclear magnetic resonance hydrogen spectroscopy (^1^H-NMR) has become one of the most reliable and suitable technologies for qualitative and quantitative comprehensive analysis due to its characteristics of being simple, uncomplicated, rapid, and nondestructive with good repeatability and stable time for sample preparation [20]. ^1^H-NMR was widely used in the structural analysis of organic compounds and their mixtures. It could clearly distinguish the chemical environment of hydrogen atoms in the sample and obtain the type and corresponding content of hydrogen atoms based on the chemical shifts and peak areas. That is, it could determine molecular structures from complex sample matrices and simultaneously quantitatively analyze multiple compounds from mixtures [21,22]. In addition, quantitative nuclear magnetic resonance spectroscopy combined with multivariate data analysis technology can simultaneously detect all active components of organic substances, which makes it possible to carry out overall identification and evaluation of asphalt aging [23,24].

**Figure 1:**
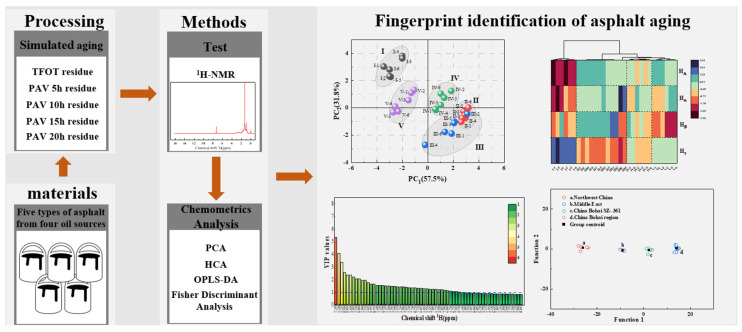
Flow chart of experimental plan procedure.

In this paper, the quantitative technology of ^1^H-NMR and multivariate data analysis were used to study the changes in chemical composition and structure of asphalt from different oil sources after aging. With this method, the whole spectrum of NMR is used as the fingerprint area without specifying specific characteristic peaks, and it has characteristics such as specificity, validity, quantifiable, stability, and reproducibility [13,25,26]. First, this study used ^1^H-NMR to identify and quantitatively analyze the fingerprints of 30 samples before and after asphalt aging from 5 different oil sources. Then, the overall differences in the asphalt structure in the aging process were analyzed through the unsupervised machine learning method. Finally, the study determined whether the “gene framework” had fundamentally changed after asphalt aging through the supervised machine learning and explored the influence of the aging process on asphalt structural change. A graphical flowchart of the experimental programs conducted in this study is shown in Figure 1.

## 2. Materials and Methods

### 2.1. Selection of Asphalt Samples

From different asphalt oil sources and manufacturers, five representative AH-70 base asphalt samples were selected. TFOT was used to carry out short-term aging on 5 kinds of base asphalts and long-term aging on PAV after TFOT aging for 5 h, 10 h, 15 h, and 20 h, and finally, 25 aged samples were obtained. Sample numbers of base asphalt and aged asphalt are shown in Table 1.

### 2.2. ^1^H-NMR Analysis

In this study, the quantitative ^1^H-NMR of asphalt samples was analyzed with a Bruker AVANCE III 600 M (Bruker, Switzerland) high-resolution NMR spectrometer. The solution was prepared at a ratio of 15 μg/500 μL (sample/solvent) using CDCl_3_ as the solvent and tetramethylsilane (TMS) as the internal standard (0.03 wt%). The test temperature was 298 K, the number of scans was 16, the number of sampling points was 32 K, and the relaxation delay time D_1_ was 10 s.

The phase, baseline, and maximum peak of the spectrum were manually corrected. Specifically, the ^1^H-NMR of each asphalt sample was imported into MestReNova 14.1 for phase correction and baseline adjustment. First, the absorption peak of the TMS internal standard was taken as the reference peak, and its chemical shift value was set to 0 ppm. Then, the absorption peak within the chemical shift of 0–10 ppm was integrated in sections, with the section interval being 0.05 ppm. Finally, the area of the absorption peak in the segmented interval was normalized, and the data matrix of each absorption peak area of all asphalt samples was obtained after removing the solvent peak CDCl_3_ and TMS peak.

### 2.3. Data Processing and Analysis

The processed ^1^H-NMR data were imported into SPSS v26.0 (IBM, Armonk, NY, USA) and SIMCA-P v14.1 (Umetrics, Umeå, Sweden) for analysis. Then PCA, HAC, OPLS-DA, and Fisher discriminant analysis were carried out on ^1^H-NMRs of asphalt samples from different oil sources before and after aging by combining unsupervised and supervised analysis methods. Among them, PCA and HAC are often used in exploratory research to visualize data sets, which are unsupervised machine learning methods. OPLS-DA and Fisher discriminant analysis are often used as algorithms for discriminant classification, which are supervised machine learning methods [27].

#### 2.3.1. Hierarchical Agglomerative Cluster (HAC)

HAC is a multivariate statistical method used to classify research samples. It can classify samples according to their closeness and similarity in nature. The ^1^H-NMR data matrix of all asphalt samples was imported into SPSS v26.0 for HAC. All asphalt samples were clustered with squared Euclidean distance by the Ward method, as shown in Equation (1). After two samples are combined, the increment of the sum of the squared deviations is regarded as the distance between clusters, and the smaller the distance, the greater the similarity between the two samples.

(1) $$D_{pq}^{2}=W_{r}-(W_{p}+W_{q})$$

In this equation, $D_{pq}^{2}$ is the distance between each class in HAC, and $W_{r}$, $W_{p}$, and $W_{q}$ are the sum of squared deviations of *r*, *p*, and *q*, respectively.

#### 2.3.2. Principal Component Analysis (PCA)

PCA is a statistical analysis method that transforms multiple indicators into a few comprehensive indicators as principal components [28]. Principal components are linear combinations of variables of the original data matrix that are orthogonal to each other and used to represent the most important information in the data matrix.

Assuming *X* = (*X*_1_, *X*_2_, …, *X_P_*)’, *p* is a random variable, and the linear variations of the principal components are as follows:

(2) $$\begin{array}{c} PC_{1}=a_{1}^{\prime }X=a_{11}X_{1}+a_{21}X_{2}+\ldots +a_{p1}X_{p}\\ PC_{2}=a_{2}^{\prime }X=a_{12}X_{1}+a_{22}X_{2}+\ldots +a_{p2}X_{p}\\ \ldots \ldots \\ PC_{p}=a_{p}^{\prime }X=a_{1p}X_{1}+a_{2p}X_{2}+\ldots +a_{pp}X_{p} \end{array}$$

The new variable *PC*_1_ is used to replace the original *p* variables *X*_1_, *X*_2_, …, *X_p_*, *PC*_1_ should reflect the original variable information as much as possible, and the second principal component *PC*_2_ can also be introduced, as can others. The main purpose of principal component analysis is to simplify data, so *m* (*m* < *p*) principal components are usually selected instead of *p* principal components in practical application. The number of principal components *m* is finally determined according to the cumulative variance contribution rate of each principal component, as shown in Equation (3).

(3) $$\mathrm{Cumulative}\;\mathrm{variance}\;\mathrm{contribution}\;\mathrm{rate}=\sum_{k=1}^{m}\lambda_{k}/\sum_{i=1}^{p}\lambda_{i}$$

In this equation, λ is the eigenvalue corresponding to each principal component; *k* is the number of selected principal components; and *i* is the total number of principal components.

The ^1^H-NMR data matrix of the obtained asphalt samples was imported into SIMCA-P14.1, and the variable of scaling type was centered. In the obtained model, R^2^ is the fitting measure, that is, the fitting degree of the model to the data, and Q^2^ represents the prediction of the model by cross-validation, that is, the accuracy of the model against the predicted new data. By this method, the redundancy and noise of ^1^H-NMR spectra are compressed and eliminated, and the evaluation results of different asphalt samples are more accurate.

#### 2.3.3. Orthogonal Partial Least Squares Discriminant Analysis (OPLS-DA)

OPLS-DA is a supervised identification model that combines orthogonal signal correction (OSC) with partial least squares discriminant analysis (PLS-DA) to effectively separate Y-predictor variables from Y-uncorrelated variables in independent variable X, as shown in Equation (4).

(4) $$X=\hat{X}+{\hat{X}}_{O}+E=TP^{T}+T_{O}P_{O}^{T}+E$$

In this equation, $T_{O}$ and $P_{O}$ are the score matrix and load matrix of Y-uncorrelated variables, respectively, identified by OSC, T and P are the score matrix and load matrix of Y-predictor variables, respectively, and E is the residual matrix. OSC filters out the variables that are not related to the category judgment and only keeps the variables that are e related to the category judgment, so that the category discriminant analysis can focus on the variables related to the category and improves the judgment ability of the pattern recognition method. The quality parameters of the OPLS-DA are R^2^X, R^2^Y, and Q^2^Y, where R^2^X and R^2^Y represent the explanatory ability of the model to the X and Y matrixes, respectively, and Q^2^Y represents the predictive ability of the model. The closer R^2^ and Q^2^ are to 1, the more stable and reliable is the model. Generally, a value higher than 0.5 indicates a good model.

#### 2.3.4. Fisher Discriminant Analysis

Fisher discriminant is one of the methods of discriminant analysis. It uses the idea of variance analysis to construct one or more linear discriminant functions y = l′x by using the p-dimensional observations of samples extracted from known populations. Let l = (l_1_, l_2_…l_p_)′, x = (x_1_, x_2_, …, x_p_)′, and the deviation between different populations (denoted as B) should be as large as possible, while the deviation within the same population (denoted as E) should be as small as possible, so as to determine the discriminant coefficient l = (l_1_, l_2_…l_p_)′. In this paper, the Fisher discriminant model of asphalt samples was established according to the principal components extracted from the PCA of the ^1^H-NMR data from the asphalt samples as the evaluation index, and the oil region number was used as the classification number.

## 3. Results and Discussion

### 3.1. ^1^H-NMR Analysis

**Figure 2:**
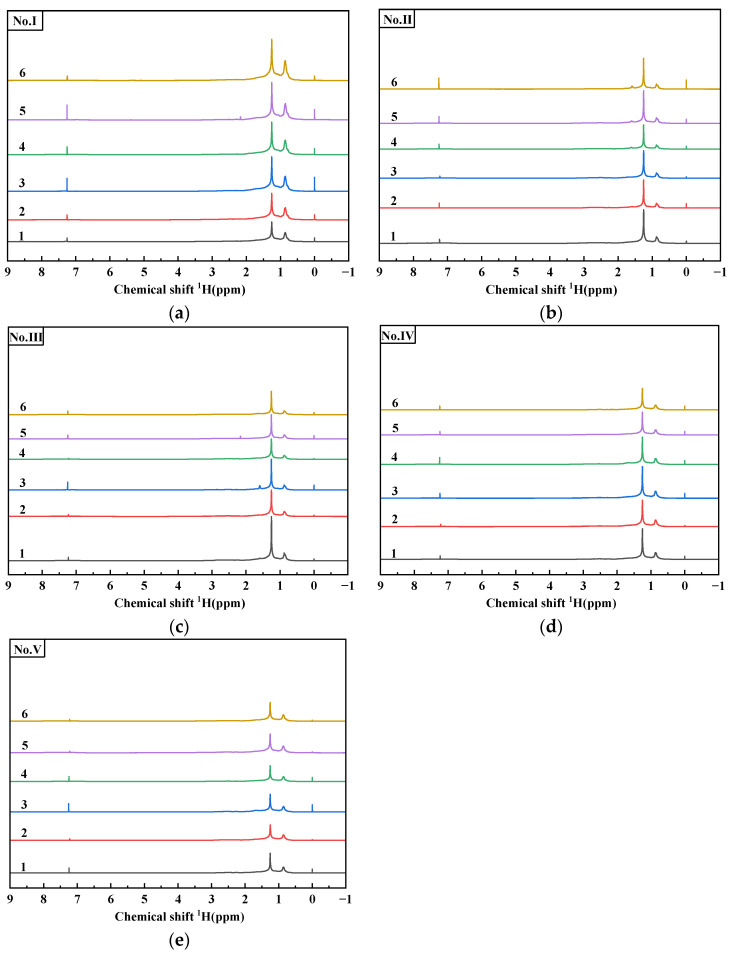
^1^H-NMR spectra of bitumen samples. (**a**) asphalt No. I; (**b**) asphalt No. II; (**c**) asphalt No. III; (**d**) asphalt No. IV; (**e**) asphalt No. V.

**Figure 3:**
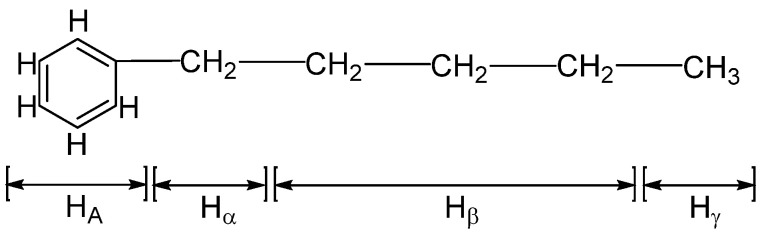
The schematic diagram of the attribution of H_A_, H_α_, H_β_, and H_γ_.

Figure 2 shows the quantitative analysis of the ^1^H-NMR of 30 asphalt samples carried out with an NMR spectrometer. In the analysis of ^1^H-NMR, the chemical composition information and the relative contents of various components in the sample can be obtained. For the attribution of different kinds of hydrogen in the ^1^H-NMR of asphalt, many researchers have investigated ion this using model compounds. At present, it is generally believed that the types of hydrogen in the spectrum can be classified into four groups: the hydrogen directly connected to aromatic-carbon (H_A_), the hydrogen connected to α carbon of the aromatic nucleus (H_α_), the hydrogen on β carbon of the aromatic nucleus and on -CH_2_-, -CH- beyond β carbon (H_β_), and the γ of the aromatic nucleus and on -CH_3_- beyond γ carbon (H_γ_) [29,30,31]. The division and attribution of ^1^H-NMR are shown in Figure 3 and Table 2.

Figure 2 shows that the ^1^H-NMR of all asphalt samples are very similar, and most of the signals overlap and are not completely distinguished. This indicates that the chemical structures of asphalt from different oil sources are highly similar, and it is reliable to use ^1^H-NMR for analysis. In the ^1^H-NMR, the hydrogen signals from strong to weak are *H_β_*, *H_γ_*, *H_α_*, *H_A_*. *H_γ_* and *H_β_* are dominant in the whole spectrum, representing the content of methyl and methylene of long-auger-chain saturated hydrocarbon in asphalt. This indicates the higher the content, the higher the saturated content in asphalt molecules.

**Figure 4:**
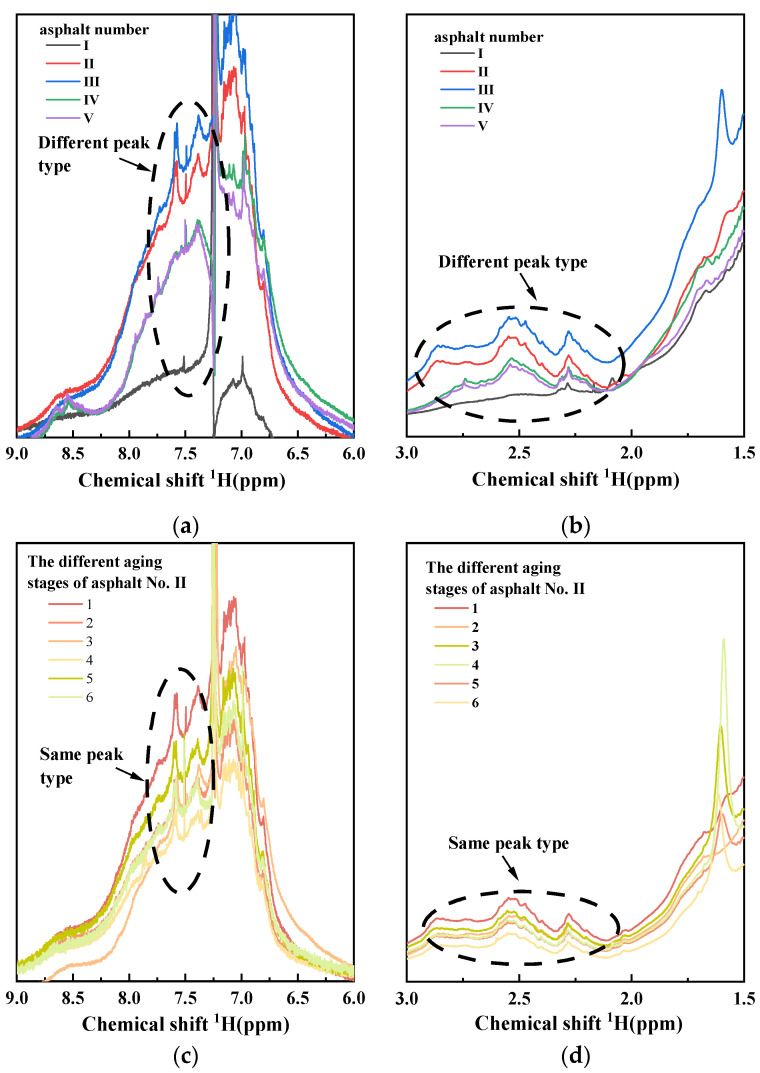
Local magnification of ^1^H-NMR. (**a**) Partial magnification of the aromatic region of the five unaged asphalts; (**b**) partial magnification of the aliphatic region of the five unaged asphalts; (**c**) partial magnification of the aromatic region of the different aging stages of asphalt No. II; (**d**) partial magnification of the aliphatic region of the different aging stages of asphalt No. II.

In addition, the difference in the hydrogen spectrum mainly lies in the aromatic region (6.0–9.0 ppm) and partial fatty region (1.5–3.0 ppm). By magnifying these two regions, it was found that the shapes of asphalt samples from different oil sources are different, as shown in Figure 4a,b. However, the spectrogram shapes of asphalt samples with different aging degrees from the same oil source are basically the same, only showing different peak intensities, as shown in Figure 4c,d. This result shows that the chemical compositions of asphalt from different oil sources are different but that aging has little effect on the chemical composition and structure of asphalt from the same oil source, and aging does not change the gene framework of asphalt.

Asphalt is a complex mixture that consists of many molecules, and it is easy to show the superposition of material signals in the ^1^H-NMR, which makes it difficult to analyze the microstructure of asphalt, which needs to be analyzed by chemometrics.

### 3.2. Principal Component Analysis (PCA)

**Figure 5:**
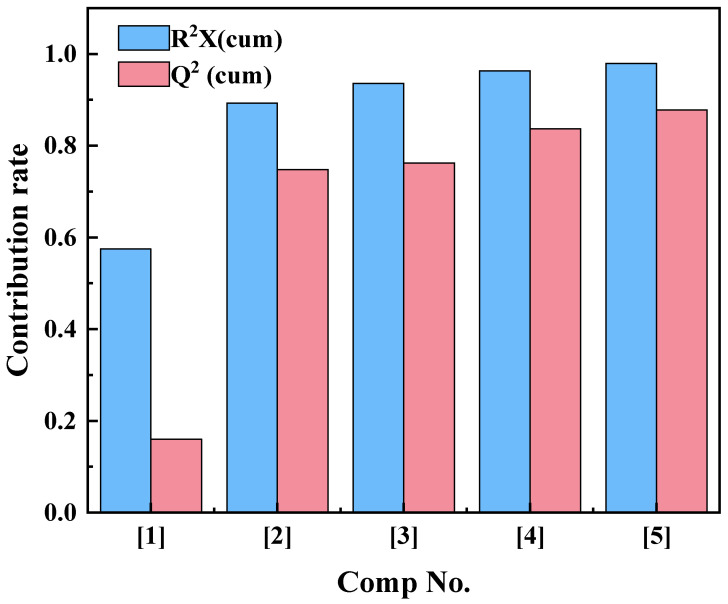
Principal component contribution of PCA analysis of asphalt samples.

PCA is a commonly used method of reducing the dimensions of a large amount of data. It can compress the original data into N principal components to describe the characteristics of the original data set and can directly reflect the differences among samples. All the data of quantitative ^1^H-NMR of 30 asphalt samples were imported into SIMCA-P14.1, and PCA was carried out to further reveal the differences of chemical composition of asphalt samples from different oil sources and with different aging degrees. Through PCA, five principal components with large contributions were extracted, as shown in Figure 5.

**Figure 6:**
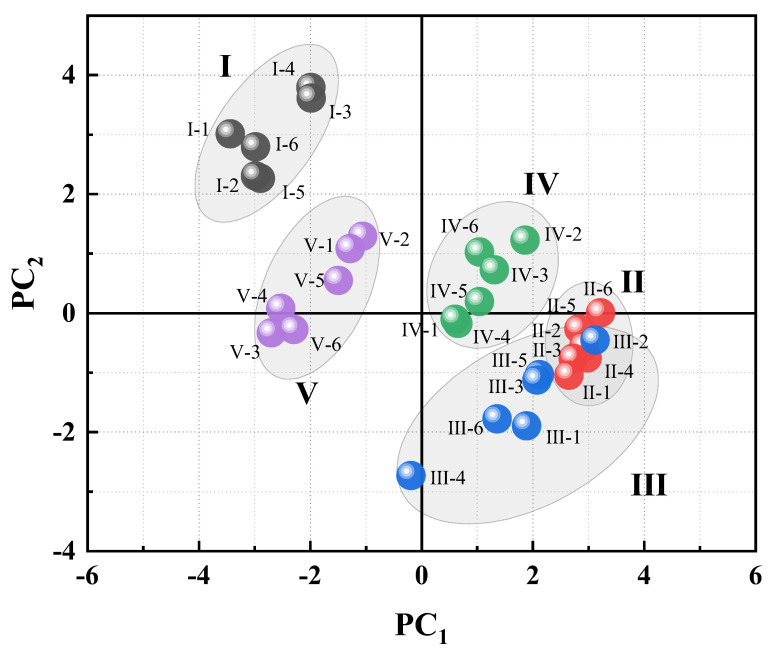
Score chart for PCA of asphalt samples.

According to this, the total variance of the first two principal components PC_1_ and PC_2_ is 89.3%, which makes a great contribution to the model and reflects that many indicators in the original data are well reflected. PC_1_ and PC_2_ are respectively taken as the X and Y axis to obtain the PCA score chart (Figure 6). The distance between two points in the chart reflects the differences in chemical composition between the two samples. The farther apart the two samples, the greater the difference in the chemical composition between them. As shown in Figure 6, the distinction between asphalt samples from different oil sources is apparently obvious, and asphalt samples from the same oil source with different aging degrees still gather together and do not cross with asphalt samples from other oil sources. This shows that the aging does not change the “gene” framework of asphalt. Among them, asphalt No. IV whose oil is from China Bohai SZ-361 gathers in the first quadrant; asphalt No. I whose oil is from northwest China and asphalt No. V from the China Bohai region both gather in the second quadrant; and asphalts No. II and No. III whose oil is from the Middle East gather in the fourth quadrant.

**Figure 7:**
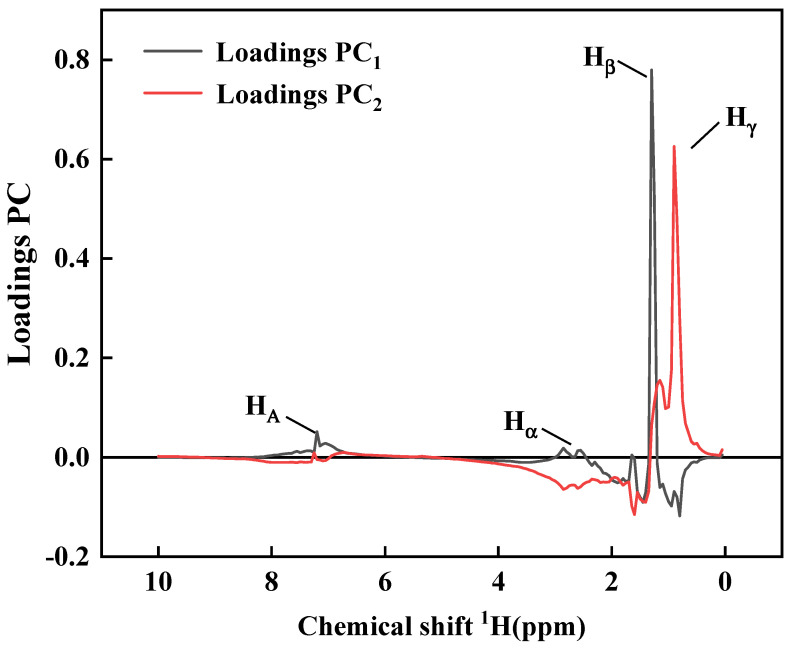
Loadings column Plot PC_1_ and PC_2_.

In order to further screen out the potential chemical structure markers that distinguish asphalt samples, the loading diagrams of the first two principal components of PCA were analyzed, and the hydrogen atoms belonging to areas related to sample clustering are pointed out in Figure 7. The chemical composition that contributes the most to the classification of different groups of samples is usually the substance that is far away from the center of the loading diagram, which shows that the farther away from the center, the greater the influence on the classification. It can be seen from the loading diagram that asphalt No. IV *H_β_*, *H_A_* in the first quadrant has a higher content, asphalts No. I and V in the second quadrant have higher contents of *H_γ_*, and asphalts No. II and No. III in the fourth quadrant may have higher contents of *H_α_*. The above results further show that the oil source of asphalt determines the chemical composition of asphalt. Although some chemical composition changes take place in the aging process of asphalt, they do not cause the fundamental changes in asphalt composition and structure. That is, the aging property of asphalt is determined by the original composition of asphalt, the oil source for producing asphalt.

### 3.3. Hierarchical Agglomerative Cluster (HAC)

**Figure 8:**
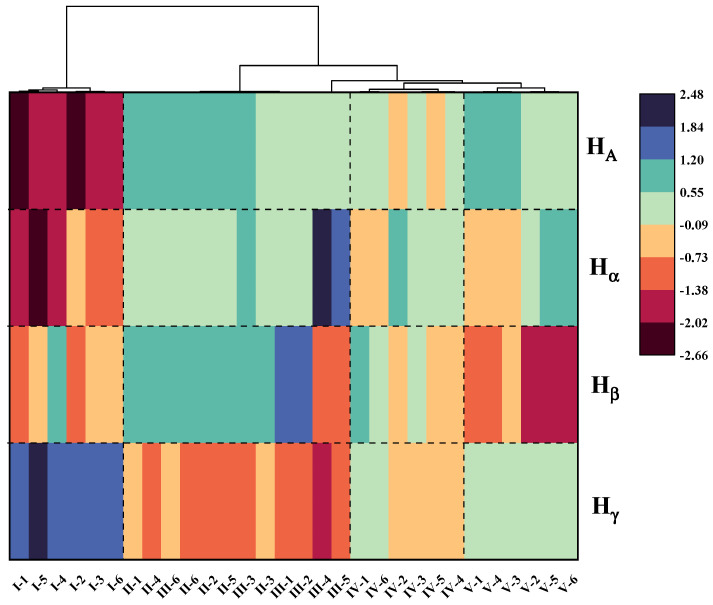
Cluster analysis pedigree chart.

In order to quantitatively analyze the differences in chemical composition among asphalt samples from different oil sources, the four hydrogen atoms belonging to areas that have the greatest influence on the clustering of asphalt oil sources were selected for cluster analysis, and a cluster heat map was drawn (Figure 8). As shown in Figure 8, the oil sources of asphalt samples can be classified according to the four kinds of hydrogen atoms. Asphalt samples from the same oil source with different aging degrees can group into one category, and the contents of the four kinds of hydrogen atoms among asphalt samples with different oil sources are obviously different. Generally, the content order of *H_γ_* is I > V > IV > II, III, *H_β_* is II, III > IV > I > V, *H_α_* is II, III > IV, V > I, and *H_A_* is II, III, V > IV > I. The results of HAC are consistent with those of PCA, which further confirms that the oil source of asphalt determines the chemical composition and aging performance of asphalt.

### 3.4. OPLS-DA Analysis

**Figure 9:**
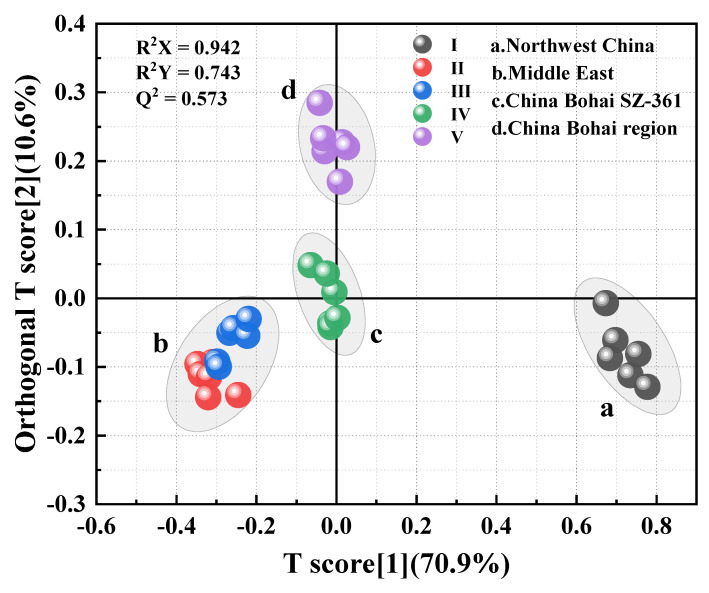
Score chart for OPLS-DA analysis of asphalt samples.

In order to further determine the differences between samples among various oil sources, OPLS-DA under the Pa scaling method was used to centralize and nondimensionalize the data [32]. Based on the principle of combining OSC with PLS, OPLS-DA with supervised pattern recognition removes the influencing factors unrelated to the classification information in the modeling process. Thus, some subtle differences among different asphalt samples can be obviously reflected, and better classification results can be obtained. As shown in Figure 9, compared with PCA, asphalt samples from different oil sources can be better separated in OPLS-DA. Through OPLS-DA, the 30 asphalt samples were divided into 4 categories according to oil source: asphalt No. I, whose oil source is northwest China, gathers in Area A; asphalts No. II and No. III, whose oil source is the Middle East, gather in Area B; asphalt No. IV, whose oil source is China Bohai SZ-361, gathers in Area C; and asphalt No. V, whose oil source is the China Bohai region, gathers in Area D. Evaluation parameters R^2^X (cum) and R^2^Y (cum) of the model, respectively, indicate the explanatory rate of the model to the X and Y matrices, and Q^2^ (cum) indicates the prediction ability of the model. In this model, Q^2^ is 0.573 > 0.5, which indicates that the model has good fit and prediction ability. In this model, the boundaries between the four types of asphalt samples are obvious and have no overlap, so the discrimination effect is good.

**Figure 10:**
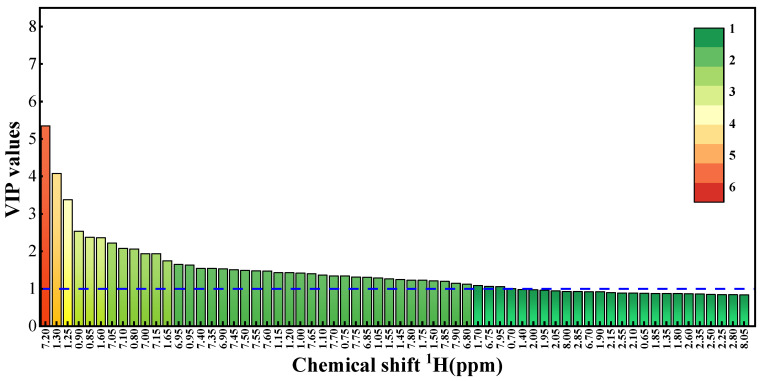
VIP values of asphalt samples.

To further judge the key chemical composition that leads to the differences in oil sources, variable importance in the projection (VIP) was used to screen the differential substances of asphalt samples from different oil sources, as shown in Figure 10. The larger the VIP, the greater the contribution of various chemical indexes to the explanatory variables, and the higher the correlation with the differences in the anti-aging performances of asphalts from different oil sources. In order to evaluate the importance of variables to the model and describe the overall contribution of each variable to the model, VIP is usually regarded as a difference substance when its threshold is greater than 1. It can be seen from the VIP diagram that the VIP of *H_A_* (7.20 ppm), *H_β_* (1.30 ppm, 1.25 ppm), and *H_γ_* (0.95 ppm, 0.85 ppm) are all greater than 1, which make great contributions. Among them, *H_A_* (7.20 ppm) which is 5.348 has the highest VIP, indicating that it is the main difference between different oil sources. The above substances are also the main chemical compositions that lead to the different anti-aging properties of asphalt samples from different oil sources. It is worth noting that the VIPs of the signals at the chemical shifts corresponding to *H_α_* (2.0–4.0 ppm) are all less than 1, which means that *H_α_* is not the differential substance between the different oil sources.

### 3.5. Fisher Discriminant Analysis

The above analysis shows that the difference between the ^1^H-NMR fingerprints of asphalt samples is determined by the oil source of asphalt, and the “gene” framework of asphalt has not changed after asphalt aging. ^1^H-NMR can be used as the basis for distinguishing the original oil source of each asphalt sample. Therefore, Fisher discriminant analysis was further used to discriminate and predict the oil source types of asphalt. In order to reduce the calculation of the model, the scores of the five principal components extracted by PCA were used as variables, and the classification results of asphalt samples obtained by PCA and OPLS-DA were further introduced into SPSS v26.0 software as grouping variables. Through step-by-step analysis, the eigenvalues of the Fisher discriminant function were output, as shown in Table 3.

As shown in Table 3, the cumulative variance contribution rate of the first two functions reached 100%, indicating that the model data obtained by PCA and PLS-DA analysis could be used to identify the five asphalt oil sources. According to the Fisher discriminant function coefficient, the discriminant functions of different oil sources were obtained as Equations (5)–(8):

(5) $$F_{a}=-101.433PC_{1}+132.216PC_{2}-33.930PC_{3}+21.244PC_{4}+13.095PC_{5}-341.202$$

(6) $$F_{b}=55.383PC_{1}-71.855PC_{2}+18.244PC_{3}-11.243PC_{4}-6.108PC_{5}-101.593$$

(7) $$F_{c}=9.033PC_{1}-12.063PC_{2}+3.283PC_{3}-1.438PC_{4}-2.060PC_{5}-4.571$$

(8) $$F_{d}=-35.271PC_{1}+45.594PC_{2}-11.497PC_{3}+6.237PC_{4}+3.363PC_{5}-42.533$$

In the equations, *F_a_*, *F_b_*, *F_c_*, and *F_d_* are respectively the discriminant scores for the northwest China, the Middle East, Bohai Suizhong 361, and Bohai region oil sources, and *PC*_1_, *PC*_2_, *PC*_3_, *PC*_4_ and *PC_5_* are the first five principal component scores in PCA. When distinguishing the oil source of the asphalt sample, the scores of the principal components corresponding to the ^1^H-NMR of the asphalt sample were entered into Equations (5)–(8) to calculate *Fa*, *Fb*, *Fc*, and *Fd*, respectively. The oil source with the highest score among these four functions is the oil source to which the asphalt to be tested belongs.

**Figure 11:**
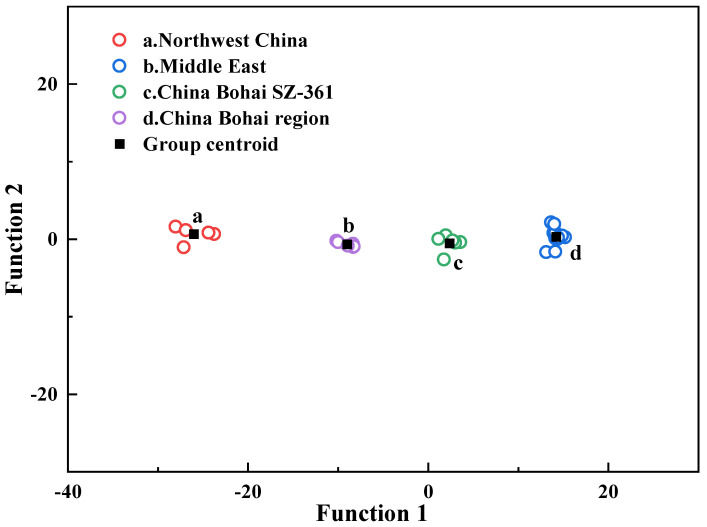
Discriminant function scatter plot.

According to the above discriminant function, all asphalt samples can be effectively discriminated. The first two discriminant functions in Table 3 were used as the plane scatter diagram of discriminant function (Figure 11). As shown in Figure 11, the 30 asphalt samples from different oil sources and with different aging degrees are accurately clustered according to oil sources, which indicates that the discrimination model is good at identifying asphalt oil sources.

In order to test the accuracy of oil source identification, the leave-one-out method was used for cross-validation. As shown in Table 4, all asphalt samples were accurately divided into four groups, and the correct rate of judgment was 100%. The results of leave-one-out cross validation further showed that the Fisher discriminant model based on ^1^H-NMR can distinguish asphalt oil sources, and it is a reliable and stable discriminant model.

## 4. Conclusions

Based on ^1^H-NMR and combined with chemometric analysis technology, this paper qualitatively and quantitatively analyzed the chemical compositions of and structural changes in asphalt from different oil sources after aging. The fingerprint identification method of asphalt aging and the discrimination model of asphalt oil source are established, and the validity of the discrimination model is verified. According to the results and discussion, the following conclusions can be drawn:

(1) Quantitative ^1^H-NMR analysis was carried out on 30 samples of 5 kinds of oil source asphalt before and after aging. The ^1^H-NMRs of the 30 asphalt samples were very similar, and hydrogen can be divided into *H_A_*, *H_α_*, *H_β_*, and *H_γ_* according to chemical shifts. The ^1^H-NMR shapes of asphalt samples from different oil sources are slightly different, while the spectrum shapes of asphalt samples from the same oil source with different aging degrees are basically the same.
(2) The quantitative ^1^H-NMRs of the 30 asphalt samples were analyzed by PCA and HAC. Asphalt samples of the same kind of asphalt and from the same kind of oil source before and after aging can be grouped into one category, whose space distance is very close. The ^1^H-NMRs of asphalt from different oil sources were far apart, and the five kinds of asphalt could be obviously grouped into four categories. The aging performance of asphalt is determined by the oil source. Although aging leads to changes in the chemical composition and structure of asphalt, it does not change the “gene framework” of asphalt.
(3) Based on ^1^H-NMR and Fisher discriminant analysis, models of four kinds of oil source asphalt after aging are established. The model data obtained by PCA and HAC can discriminate the asphalt from different kinds of oil sources, and the established Fisher discriminant function model has an accuracy of 100%.

## Figures and Tables

**Table 1:** Asphalt oil source and asphalt sample code.

Base Asphalt	TFOT	PAV 5 h	PAV 10 h	PAV 15 h	PAV 20 h	Oil Source
I-1	I-2	I-3	I-4	I-5	I-6	a. Northwest China (China Karamay)
II-1	II-2	II-3	II-4	II-5	II-6	b. Middle East (Korea Ssangyong)
III-1	III-2	III-3	III-4	III-5	III-6	b. Middle East (China Qilu)
IV-1	IV-2	IV-3	IV-4	IV-5	IV-6	c. China Bohai SZ-361 (China Zhonghai SZ-361)
V-1	V-2	V-3	V-4	V-5	V-6	d. China Bohai region (China Liaohe)

**Table 2:** Types of protons in the ^1^H-NMR spectrum.

Signal	Chemical Shift δ, ppm (Base on TMS)	Types of Protons
*H* *_γ_*	0.5~1.0	Hydrogen linked to γ-carbon of the aromatic nucleus and γ beyond CH_3_, CH group
*H* *_β_*	1.0~2.0	Hydrogen linked to β-carbon of the aromatic nucleus and β beyond CH_2_, CH group
*H* *_α_*	2.0~4.0	Hydrogen linked to α-carbon of the aromatic Nucleus
*H* *_A_*	6.0~9.0	Hydrogen directly linked to aromatic carbon

**Table 3:** Fisher discriminant function eigenvalues.

Function	Eigenvalue	Variance / %	Cumulative/%
1	253.161	99.8	99.8
2	0.308	0.2	100.0
3	0.268	0.0	100.0

**Table 4:** Original and cross verification of Fisher’s discrimination function.

Count		Group	Classification results Predication group members				Total
			1	2	3	4	
**Cross validation**	**Count**	1	5	0	0	0	5
		2	0	12	0	0	12
		3	0	0	6	0	6
		4	0	0	0	6	6
	%	1	100.0	0.0	0.0	0.0	100.0
		2	0.0	100.0	0.0	0.0	100.0
		3	0.0	0.0	100.0	0.0	100.0
		4	0.0	0.0	0.0	100.0	100.0

## Data Availability

Not applicable.
